# Investigating molecular transport in the human brain from MRI with physics-informed neural networks

**DOI:** 10.1038/s41598-022-19157-w

**Published:** 2022-09-14

**Authors:** Bastian Zapf, Johannes Haubner, Miroslav Kuchta, Geir Ringstad, Per Kristian Eide, Kent-Andre Mardal

**Affiliations:** 1 Faculty of Mathematics and Natural Sciences, University of Oslo, 0851 Oslo, Norway; 2 Department of Numerical Analysis and Scientific Computing, Simula Research Laboratory, 0164 Oslo, Norway; 3 Department of Radiology, Oslo University Hospital, 0372 Oslo, Norway; 4 Department of Geriatrics and Internal medicine, Sorlandet Hospital, 4838 Arendal, Norway; 5 Department of Neurosurgery, Oslo University Hospital, 0372 Oslo, Norway; 6 Institute of Clinical Medicine, University of Oslo, 0372 Oslo, Norway

**Keywords:** Biophysical models, Applied mathematics

## Abstract

In recent years, a plethora of methods combining neural networks and partial differential equations have been developed. A widely known example are physics-informed neural networks, which solve problems involving partial differential equations by training a neural network. We apply physics-informed neural networks and the finite element method to estimate the diffusion coefficient governing the long term spread of molecules in the human brain from magnetic resonance images. Synthetic testcases are created to demonstrate that the standard formulation of the physics-informed neural network faces challenges with noisy measurements in our application. Our numerical results demonstrate that the residual of the partial differential equation after training needs to be small for accurate parameter recovery. To achieve this, we tune the weights and the norms used in the loss function and use residual based adaptive refinement of training points. We find that the diffusion coefficient estimated from magnetic resonance images with physics-informed neural networks becomes consistent with results from a finite element based approach when the residuum after training becomes small. The observations presented here are an important first step towards solving inverse problems on cohorts of patients in a semi-automated fashion with physics-informed neural networks.

## Introduction

In the recent years there has been tremendous activity and developments in combining machine learning with physics-based models in the form of partial differential equations (PDE). This activity has lead to the emergence of the discipline “physics-informed machine learning”^1^. Therein, nowadays, arguably one of the most popular approaches are physics-informed neural networks (PINNs)^2–4^. They combine PDE and boundary/initial condition into a non-convex optimization problem which can be implemented and solved using mature machine learning frameworks while easily leveraging modern hardware (e.g. GPU-accelerators). One of the benefits of the PINN compared to traditional numerical methods for PDE is that no computational mesh is required. Further, inverse PDE problems are solved in the same fashion as forward problems in PINNs. The only modifications to the code are to add the unknown PDE parameters one seeks to recover to the set of optimization parameters and an additional data-discrepancy term to the objective function. The PINN training process, however, is challenging and can require significant computing resources. Several works have put forward approaches to address this issue, among them extreme learning machines^5^, importance sampling^6^ and adaptive activation functions^7^. Another challenge in training PINNs is balancing boundary, initial and PDE loss terms. This challenge has been addressed by adaptive weighting strategies^8–11^, as well as theory of functional connections^12,13^. Despite these challenges, the effectiveness of the method has been demonstrated in a wide range of works, examples include turbulent flows^14^, heat transfer^15^, epidemiological compartmental models^16^ or stiff chemical systems^17^.

Among other approaches^18–20^, PINNs can be used to discover unknown physics from data. In the context of computational fluid dynamics, PINNs have been successfully applied in inverse problems using simulated data, see, e.g.,^14,21–24^ and real data^25,26^. A comprehensive review on PINNs for fluid dynamics can be found in^27^.

In this work, we solve an inverse biomedical flow problem in 4D with unprocessed, noisy and temporally sparse MRI data on a complex domain. Classical approaches require careful meshing of the brain geometry and making assumptions on the boundary conditions^28^. In patient-specific brain modeling the meshing is particularly challenging and requires careful evaluation of the generated meshes^29^. Physics-informed neural networks have been applied for the discovery of unknown physics from data without meshing and without regularization^3^. This makes the PINN method an appealing and promising approach that avoids major challenges in our application and is therefore well worth investigation. However, PINNs introduce other challenges such as the choice of the network architecture, the optimization algorithm and hyperparameter tuning, e.g., weight factors in the loss function. Nevertheless, it is worth to examine how PINNs perform compared to classical algorithms in our application.

We aim to perform a computational investigation of the glymphatic theory based on and similar to^28,30^ with PINNs. We apply them to model the fluid mechanics involved in brain clearance. Various kinds of dementia have recently been linked to a malfunctioning waste-clearance system - the so-called glymphatic system^31^. In this system, peri-vascular flow of cerebrospinal fluid (CSF) plays a crucial role either through bulk flow, dispersion or even as a mediator of pressure gradients through the interstitium^32^. While imaging of molecular transport in either rodents^33^ or humans^34^ points towards accelerated clearance through the glymphatic system, the detailed mechanisms involved in the system are currently debated^35–40^.

Our approach builds on previous work where the estimated apparent diffusion coefficient (ADC) for the distribution of gadobutrol tracer molecules over 2 days, as seen in T1-weighted magnetic resonance images (MRI) at certain time points, is compared with the ADC estimated from diffusion tensor images (DTI)^28^. The ADC of gadobutrol was estimated from the T1-weighted images based on simulations using the finite element method (FEM) for optimal control of the diffusion equation. The findings were then compared to estimates of the apparent diffusion coefficient based on DTI. The latter is a magnetic resonance imaging technique that measures the diffusion tensor of water on short time scales, which in turn can then be used to estimate the diffusion tensor for other molecules, such as gadobutrol^28^. The limited amount of available data prevents from quantifying the uncertainty in the recovered parameters, and makes it a challenging test case for comparing PINNs and finite element based approaches.

Among other works involving physics-informed neural networks and MRI data^41,42^ several works have previously demonstrated the effectiveness of PINNs in inverse problems related to our application. PINNs have been applied to estimate physiological parameters from clinical data using ordinary differential equation models^43^, but we here consider a PDE model. Parameter identification problems involving MRI data and PDE have been solved using PINNs^26,44^, but the geometries are reduced to 1-D and hence, taking into account the time dependence of the solution, an effectively two-dimensional problem is solved. Both approaches further involve a data smoothening preprocessing step.

To the best of our knowledge, this work is the first to estimate physiological parameters from temporally sparse, unsmoothened MRI data in a complex domain using a 4-D PDE model with PINNs. We start to verify the PINNs approach on carefully manufactured synthetic data, before working on real data. The synthetic testcases reveal challenges that occur for the PINNs due to noise in the data and the sensitivity of the neural network training procedure to different choices of hyperparameters. For all of the chosen hyperparameter settings, we evaluate the accuracy of the recovered diffusion coefficient based on the value of the PDE and data loss. For the synthetic test case, as well as for the real test case, it is required to ensure vanishing PDE loss in order to be consistent with the finite element approach. The question on how this is achieved is addressed by heuristics. We investigate using the $\ell ^1$-norm instead of $\ell ^2$-norm for the PDE loss as an alternative to avoid the overfitting. We further discuss how to solve additional challenges that arise when applying the PINNs to real MRI data. Throughout the paper, we solve the problem with both PINNs and FEM.

**Figure 1:**
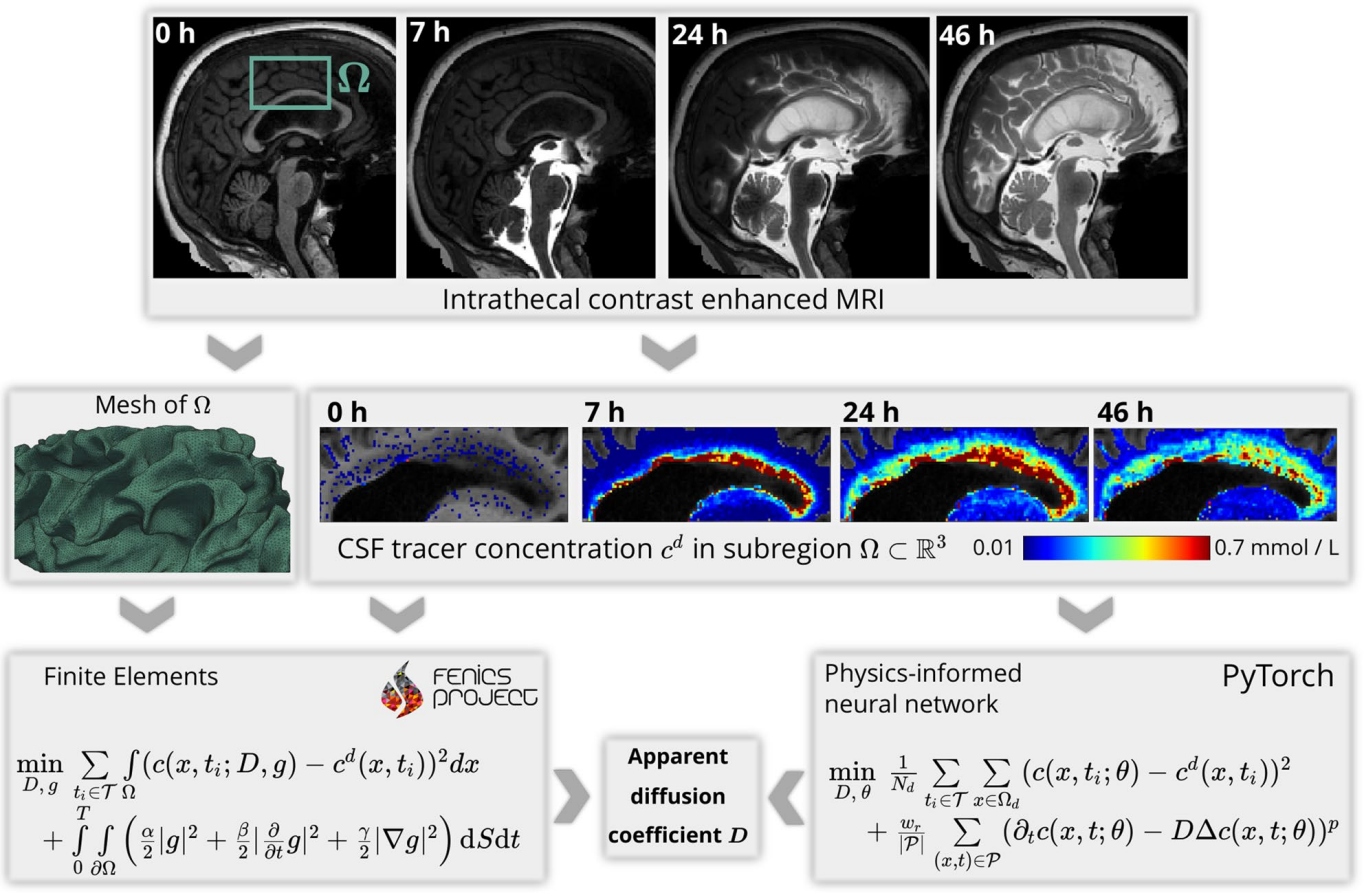
Flowchart illustrating our workflow from clinical images to estimated tracer diffusivity in the human brain. From the FreeSurfer^45^ segmentation of a baseline MRI at $t=0$, we define and mesh a subregion $\Omega$ of the white matter. Intrathecal contrast enhanced MRI at later times $t=7,\, 24,\, 46$ h are used to estimate the concentration of the tracer in the subregion. We then use both a finite element based approach and physics-informed neural networks to determine the scalar diffusion coefficient that describes best the concentration dynamics in $\Omega$.

## Problem statement

Given a set of concentration measurements $c^d(x_j, t_i)$ at four discrete time points $t_i \in \{ 0, 7, 24, 46 \}$ h and voxel center coordinates $x_j \in \Omega$, where $\Omega \subset {\mathbb {R}}^3$ represents a subregion of the brain, we seek to find the apparent diffusion coefficient $D>0$ such that a measure $J(c, c^d)$ for the discrepancy to the measurement is minimized under the constraint that *c*(*x*, *t*) fulfills

1 $$\begin{aligned} \frac{\partial }{\partial t} c= D \Delta c \quad \text {in } \Omega \times (0,T). \end{aligned}$$

The apparent diffusion coefficient takes into account the tortuosity $\lambda$ of the extracellular space of the brain and relates to the free diffusion coefficient $D_f = \lambda ^2 D$^46^. Similar to Valnes et al.^28^ we here make the simplifying assumption of a spatially constant scalar diffusion coefficient. Diffusion of molecules in the brain matter is known to be anisotropic^46,47^. In Supplementary Section S2 online we assess the anisotropy in the white matter for the patient under consideration in this work. The fractional anisotropy is $0.27 \pm 0.15$ in $\Omega$, indicating that molecular diffusion is rather isotropic. Moreover, we show there that simulations based on anisotropic, inhomogeneous DTI are, up to relative error of $9\,\%$, comparable to simulations based on the patient-specific isotropic, homogeneous mean diffusion coefficient. This serves as justification for the simplifying assumption of a constant diffusion coefficient used in this work. The initial and boundary conditions required for the PDE (1) to have a unique solution are only partially known, and the differing ways in which we choose to incorporate them into the the PINN and FEM approaches are described in sections “The PINN approach” and “The finite element approach”.

**Figure 2:**
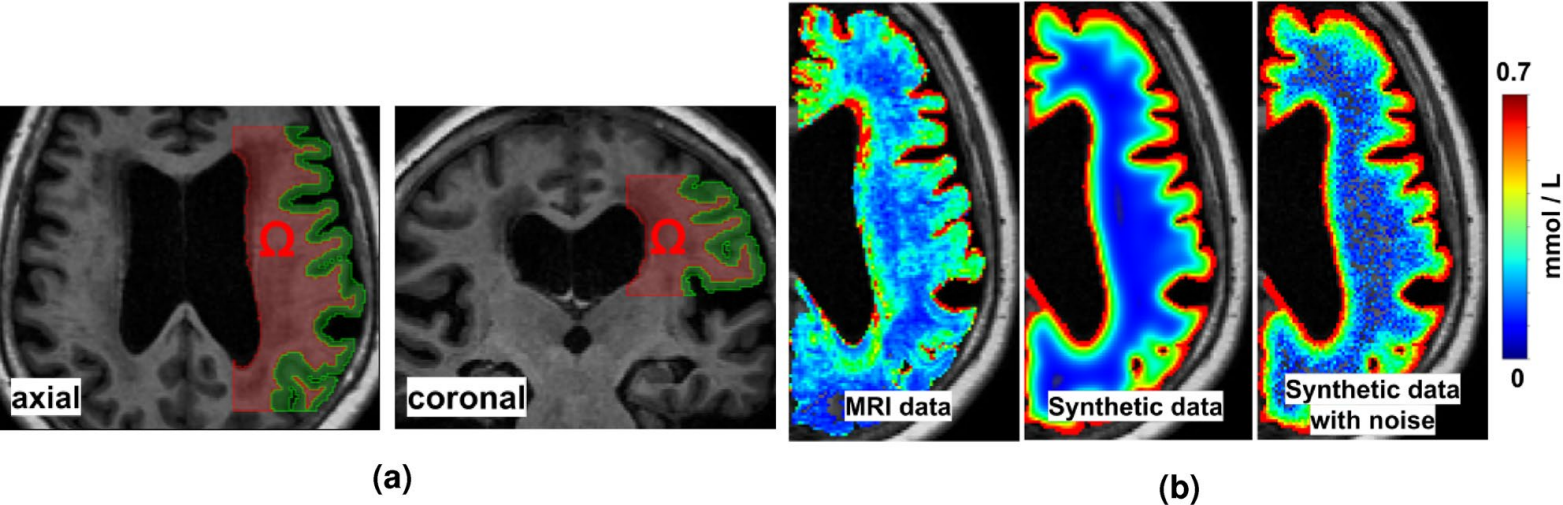
Geometries and data considered in this work. (**a**) Axial and coronal slices through the subregion $\Omega$ of the white matter we consider in this work. The green region depicts the gray matter and is drawn to illustrate the geometrical complexity of the grey matter. (**b**) Axial view of the tracer concentration after 24 h in the right hemisphere for the three data sets under consideration. Note how the tracer enters the brain from CSF spaces (black).

Our workflow to solve this problem on MRI data is illustrated in Fig. 1. Figure 2a illustrates the white matter subregion $\Omega \subset {\mathbb {R}}^3$ we consider in this work. Figure 2b shows a slice view of the concentration after 24 h for the three datasets considered in this work, i.e., MRI data, synthetic data with and without noise. In all cases, we use data at ${\mathcal {T}} = \{ 0, 7, 24, 46\}$ h (after tracer injection at $t=0$).

## Results

### Synthetic data

We first validate the implementation of both approaches by recovering the known diffusion coefficient $D_0$ from synthetic data without noise. We find that both approaches can be tuned to recover the diffusion coefficient to within a few percent accuracy from three images. Further details can be found in Supplementary Section S4 online.

### Synthetic data with noise

We next discuss how to address challenges that arise for our PINN approach when trained on noisy data as specified by Supplementary Equation (S2). We find (see Supplementary Table S2 online for the details) that smaller batch sizes of $\sim \, 10^4$ points per loss term result in more accurate recovery of the diffusion coefficient (for fixed number of epochs). We hence divide data and PDE points into 20 batches with $1.5 \times 10^4$ and $5 \times 10^4$ samples per batch, respectively, for the following results. In all the results with synthetic data reported in this work, we trained the PINN for 20,000 epochs.

**Figure 3:**
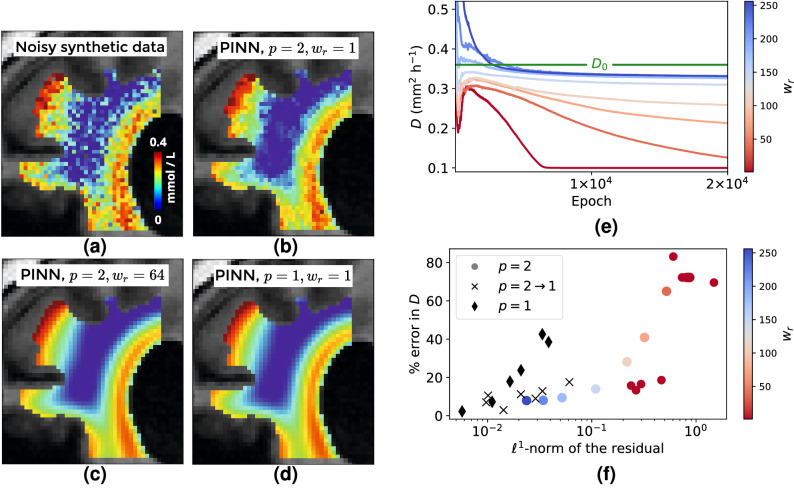
Influence of PINN hyperparameters on the diffusion coefficient estimated from noisy synthetic data. (**a**) Coronal slice of synthetic data with noise after $46\,$h, compared to predictions $c(x,t=46\, \mathrm {h}, \theta _{\mathrm {opt}})$ of trained PINN models with different hyperparameters in the loss function (4). The overfitting seen in the PINN with $p=2, w_r=1$ (**b**) can be prevented by using either increased PDE weight $w_r$ (**c**) or the $\ell ^1$-norm for the PDE loss (**d**). (**e**) The diffusion coefficient recovered by the PINN trained on noisy synthetic data converges to $D_{\mathrm {min}}$ for PDE weight $w_r \le 2$ in the loss function (4). (**f**) Relative error in recovered *D* from noisy synthetic data as a function of the residual after training for the results presented in (**e**) and Table 1. Color encodes the PDE weight $1 \le w_r \le 256$ for the results with $p=2$ (dotted). Black markers indicate results with either switching $p=2\rightarrow 1$ during training or $p=1$. Different hyperparameter settings in the PINN loss (4) yield models which fulfill the PDE to different accuracy, and low values for the residual coincide with more accurate recovery of the diffusion coefficient.

In Fig. 3a,b we compare the data to output of the PINN after training with the ADAM optimizer^48^ and exponential learning rate decay from $10^{-3}$ to $10^{-4}$ for $2\times 10^4$ epochs. In detail, after training we use the PINN as a forward surrogate model with the optimized weights and biases $\theta _{\mathrm {opt}}$ to compute the output $c(x,t; \theta _{\mathrm {opt}})$ at time *t* and voxel coordinates *x*.

The figures indicate that the network is overfitting the noise that was added to the synthetic data. This in turn leads to the diffusion coefficient converging to the lower bound $D_{\mathrm {min}}=0.1$ $\hbox {mm}^2\,\hbox {h}^{-1}$ during optimization as shown in Fig. 3e. Here we discuss two remedies: (i) increasing the regularizing effect of the PDE loss via increasing the PDE weight $w_r$ and (ii) varying the norm in the PDE loss. We observe from Fig. 3e that for $w_r \gtrsim 64$ the recovered *D* converges towards the true value to within $\approx 10 \, \%$ error. It can also be seen that increasing the weight further does not significantly increase the accuracy. Figure 3b,c show the predicted solution after 46 h of the trained PINN. It can be seen that the overfitting occurring for $w_r=1$ is prevented by choosing a $w_r \ge 64$. These results are in line with the frequent observation that the weights of the different loss terms in PINNs are critical hyperparameters. Since we assume that the data is governed by a diffusion equation (with unknown diffusion coefficient), we want the PDE residual to become small. As demonstrated above, this can be achieved by increasing the PDE weight. The correlation between a large weight, a low PDE residual and a more accurate recovery of the diffusion coefficient is visualized in Fig. 3f.

Figure 3f also demonstrates the effectiveness the strategy (ii) to successfully lower the PDE residual, which is based on using the $\ell ^1$-norm for the PDE loss. Using this norm makes the cost function less sensitive to outliers in the data where the observed tracer distribution $c^d$ deviates from the diffusion model (1).

Exemplarily, we demonstrate the effectiveness of this approach in Fig. 3d. There, we plot the PINN prediction after training with $p=1$. It can be seen that the prediction is visually identical to the prediction obtained with $p=2$ and $w_r=64$ (The relative difference between the predictions in Fig. 3c,d is about 2 %).

The results in Fig. 3f are obtained in a systematic study with fixed $w_r=1$. In detail, we test the combinations of the following hyperparameters:

- Parameterizations $D(\delta )$ (10) vs. $D = \delta$ (11) of the diffusion coefficient in terms of a trainable parameter $\delta$, c.f. section “Parameterization of the diffusion coefficient”
- $p=1$, switching $p=2 \rightarrow 1$ after half the epochs, $p=2$
- fixed learning rate $10^{-3}$, exponential learning rate decay $10^{-3} \rightarrow 10^{-4}$, fixed learning rate $10^{-4}$ and exponential learning rate decay $10^{-4} \rightarrow 10^{-5}$.

Table 1 reports the relative error in the recovered diffusion coefficient after $2 \times 10^4$ epochs of training with ADAM and the minibatch sampling described in Supplementary Algorithm 1 online. From the table it can be observed that for $D=\delta$ and $p=1$ instabilities occur with the default learning rate $10^{-3}$ and, due to exploding gradients, the algorithm fails. This problem does not occur when using the parameterization $D=D(\delta )$ (10). It can further be observed that both parameterizations can be fine tuned to achieve errors $\lesssim 10\%$ in the recovered *D*. However, the table shows that it is *a priori* not possible to assess which hyperparameter performs best since, for example, settings that fail for the parameterization $D = \delta$ (11) work well with $D(\delta )$ (10).

We hence investigate the effect of the different hyperparameters on the trained PINN and compute the $\ell ^1$-norm of the residual after training defined as

2 $$\begin{aligned} \frac{1}{{|{\mathcal {P}}_{\tau }|}} \sum \limits _{(x,t) \in {\mathcal {P}}_{\tau }} \left| \partial _t c(x,t; \theta ) -D \Delta c(x,t; \theta ) \right| . \end{aligned}$$

Here, ${\mathcal {P}}_{\tau } = \tau \times \Omega _p$, where $\tau = \{0, \ldots , T\}$ are 200 linearly spaced time points between first and final image at $T=46\,$h and $\Omega _r$ denotes the set of center coordinates of all the voxels inside the PDE domain. Note that we evaluate (2) with the recovered diffusion coefficient, not with the true $D_0$. Table 1 also reports this norm for the different hyperparameter settings. It can be seen that different hyperparameters lead to different norms of the PDE residual. Table 1 reveals that low values of the residual correspond to more accurate recovery of the diffusion coefficient. These results are plotted together with the results from Fig. 3e in Fig. 3f where it can be seen that low PDE residual after training correlates with more accurate recovery of the diffusion coefficient. This underlines our observation that it is important in our setting to train the PINN such that the norm of the PDE residual is small.

Finally, for the FEM approach, Supplementary Table S4 online tabulates the relative error in the recovered diffusion coefficient for solving (7) with regularization parameters spanning several orders of magnitude. Similar to the PINN results, the parameterization $D=D(\delta )$ (10) can avoid numerical instabilities. As with the PINN approach, the FEM approach yield estimates of the diffusion coefficient accurate to $\lesssim 10\%$ for proper choice of regularization parameters. The results are in line with the well-established observation that a sophisticated decrease of the noise level and regularization parameters ensures convergence towards a solution^49^.

**Table 1:** Rel. error $|D-D_0|/D_0$ in % in the diffusion coefficient and PDE residual norm after training (in brackets) for different optimization strategies averaged over 4 trainings on synthetic data with noise. It can be seen that the accuracy correlates with the PDE residual after training, i.e. the lower the PDE residual, the more accurate the recovered diffusion coefficient. This relation is further illustrated in Fig. 3f. Failure of the algorithm is indicated by the symbol “$\times$”.

Parameterization	*p*	lr			
		$10^{-3}$	$10^{-3}$ $\rightarrow 10^{-4}$	$10^{-4}$	$10^{-4}$ $\rightarrow 10^{-5}$
$D=\delta$	1	$\times$	$\times$	18 (1.6e−02)	43 (3.4e−02)
	$2\rightarrow 1$	$\times$	7 (9.7e−03)	3 (1.4e−02 )	13 (3.4e−02)
	2	70 (1.5e+00)	83 (6.1e−01)	16 (2.4e−01)	17 (3.0e−01 )
$D=D(\delta )$	1	7 (1.1e−02)	2 (5.7e−03)	24 (2.1e−02)	39 (3.9e−02)
	$2\rightarrow 1$	11 (2.1e−02)	11 (1.0e−02 )	9 (2.9e−02)	18 (6.1e−02)
	2	72 (7.3e−01)	72 (7.7e−01)	13 (2.7e−01 )	19 (4.7e−01)

### MRI data

We proceed to estimate the apparent diffusion coefficient governing the spread of tracer as seen in MRI images. It is worth emphasizing here that our modeling assumption of tracer transport via diffusion with a constant diffusion coefficient $D \in {\mathbb {R}}$ is a simplification, and that we can not expect perfect agreement between model predictions and the MRI data. Furthermore, closer inspection of the tracer distribution on the boundary in Fig. 2b reveals that, unlike in the synthetic data, the concentration varies along the boundary in the MRI measurements. Based on these two considerations it is to be expected that challenges with the PINN approach arise that were not present in the previous, synthetic testcases. However, our previous observation that smaller PDE residual correlates with more accurate recovery of the diffusion coefficient serves as a guiding principle on how to formulate and minimize the PINN loss function such that the PDE residual becomes small.

Based on the observation that the parameterization $D=D(\delta )$ avoids instabilities during the optimization, we only use this setting in this subsection. The white matter domain $\Omega$ is the same as in the previous section, and we again divide both data and PDE loss into 20 minibatches. We train for $10^5$ epochs using the ADAM optimizer with exponentially decaying learning rate $10^{-4}$ to $10^{-5}$. The reason we have to train the PINN for more epochs on MRI data compared to the synthetic test case (where we used 20,000 epochs) is the need for using lower learning rate together with learning rate decay to avoid convergence into a bad local minimum (where typically $c(x,t; \theta )=\mathrm {const}$ and $D\rightarrow 0$).

**Figure 4:**
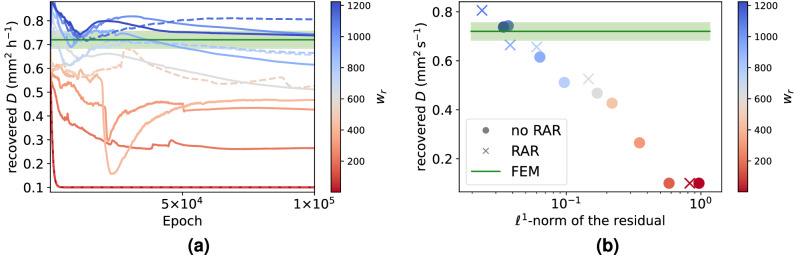
Influence of PINN hyperparameters on the diffusion coefficient estimated from clinical data. (**a**) Diffusion coefficient during training for different PDE weights $w_r$ and exponentially decaying learning rate from $10^{-4}$ to $10^{-5}$. Dashed lines indicate result with residual based adaptive refinement (RAR). (**b**) Estimated diffusion coefficient with $p=2$ for different PDE weights $w_r$ as a function of the $\ell ^1$-norm of the residual after training. The values for FEM and the green horizontal bars indicating an error estimate are taken from Valnes et al.^28^.

We first test for $p=2$ with PDE weight $w_r \in \{1, 32, 64, 128, 256, 512, 1024 \}$ and display the results in Fig. 4a. It can be seen that, similar to the noisy synthetic data, the diffusion coefficient converges to the lower bound for low PDE weights. For these settings, we plot the residual norm (2) of the trained networks in Fig. 4b. It can be seen that increased PDE weight leads to lower residual after training, and in turn to an estimate for *D* which becomes closer to FEM.

**Figure 5:**
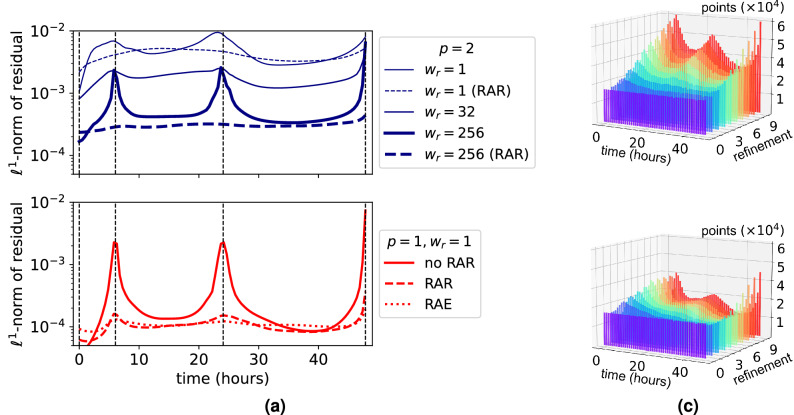
Adaptive training point refinement is needed to fulfill the PDE in all timepoints. (**a**) Average PDE residual in $\Omega _P$ over time for different optimization schemes. Vertical lines (dashed) indicate the times where data is available. In all cases, the learning rate decays exponentially from $10^{-3}$ to $10^{-4}$. (**b**,**c**) Distribution of PDE points during training with RAR (**b**) and RAE (**c**). Starting from a uniform distribution of points (in time), more points are added at 7, 24 and 46 h where data is available.

Further, in Fig. 5a we also plot the $\ell ^1$-norm of the residual after training as a function of time $t\in [0, T]$, defined as

3 $$\begin{aligned} r(t) = \frac{1}{|\Omega _r|} \sum \limits _{x \in \Omega _r} \left| \partial _t c(x,t; \theta ) -D \Delta c(x,t; \theta ) \right| . \end{aligned}$$

The continuous blue lines in Fig. 5a exemplarily show *r*(*t*) for some PDE weights. It can be seen that higher PDE weights lead to lower residuals. However, for $w_r = 256$ the PDE residual is significantly higher at the times where data is available than in between. We did not observe this behavior in the synthetic testcase. Since we want the modeling assumption (1) to be fulfilled equally in $\Omega \times [0, T]$, we use residual based adaptive refinement (RAR)^50^. Using the RAR procedure, we add $10^5$ space-time points to the set ${\mathcal {P}}$ of PDE points after $1\times 10^4,\, 2\times 10^4, \ldots ,\, 9\times 10^4$ epochs. Details on our implementation of RAR and an exemplary loss plot during PINN training are given in Supplementary Section S5.2 online. The effectiveness of RAR to reduce this overfitting is indicated by the dashed blue lines in Fig. 5a.

**Figure 6:**
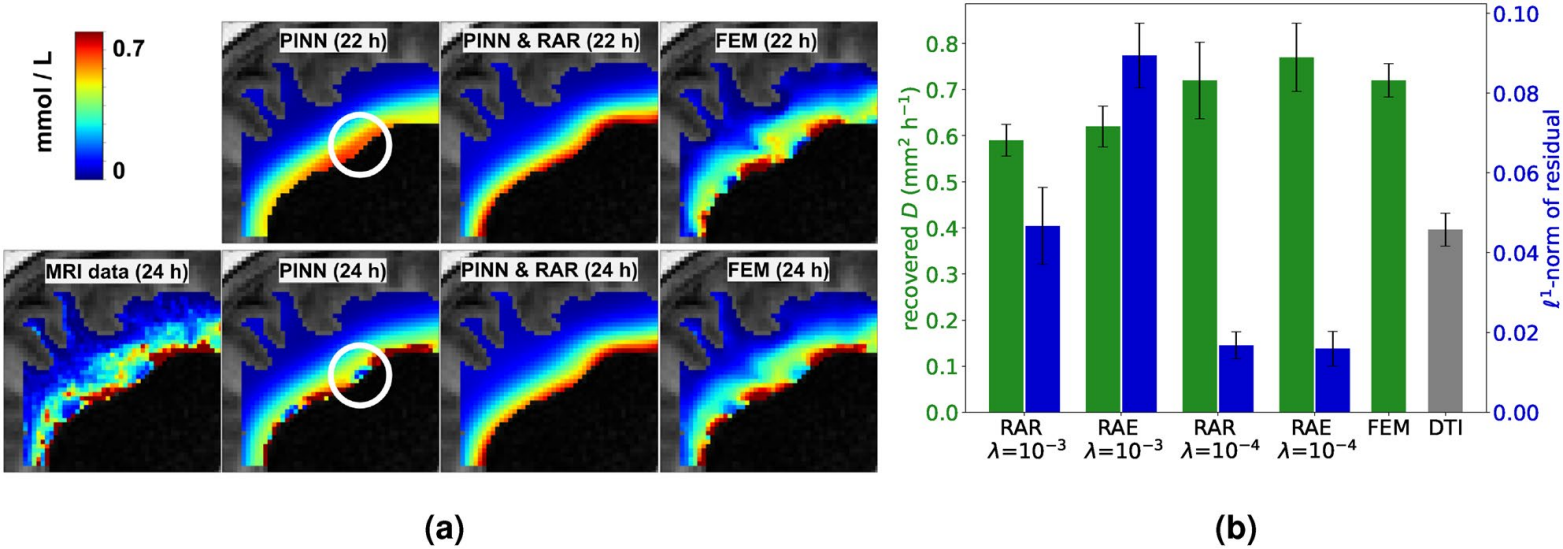
Adaptive refinement yields PINN solutions that are consistent with a diffusion model. (**a**) Upper row: Output $c(x,t=22\, \mathrm {h}, \theta _{\mathrm {opt}})$ of PINNs models trained with $p=1$ and $p=1$ & RAR and FEM solution for $(\alpha , \beta , \gamma ) = (10 ^{-6}, 0.1, 0.01)$. Lower row: Zoom into a sagittal slice of data at $24\,$h compared PINN and FEM solutions. The PINN prediction after training without RAR overfits the data. Compare also to Fig. 5. (**b**) Green: PINN estimates for the diffusion coefficient with RAR or RAE and different initial learning rates ($p=1$ in all cases). Blue: $\ell ^1$-norm of the residual after training. It can be seen that lower learning rate leads to a lower residual norm and an estimate for the diffusion coefficient closer to the FEM approach.

Next, we test for $p=1$ with an exponentially decaying learning rate from $10^{-3}$ to $10^{-4}$ as well as $10^{-4}$ to $10^{-5}$. With this setting, the PINNs approach yields an estimate $D= 0.75$ $\hbox {mm}^2\,\hbox {h}^{-1}$ which is close to the FEM solution^28^ $D = 0.72$ $\hbox {mm}^2\,\hbox {h}^{-1}$. However, a closer inspecting of the PINN prediction at 22 and 24 (where data is available) shown in Fig. 6a reveals that the PINN is overfitting the data. This is further illustrated by the continuous red line in Fig. 5 where it can be seen that the PDE residual is one order of magnitude higher at the times where data is available. The dashed red line in Fig. 5 and slices of the predicted $c(x,t;\theta _{\mathrm {opt}})$ shown in Fig. 6a show that this behavior can be prevented by using RAR. The FEM approach also shown in Fig. 6a resolves the boundary data in more detail than the PINN solution obtained with RAR. This can be explained by the fact that the boundary condition *g* explicitly enters the FEM approach as a control variable.

Since the RAR procedure increases the number of PDE points, the computing time increases (by about 25 % in our setting). We hence test a modification of the RAR procedure. Instead of only adding points, we also remove the points from ${\mathcal {P}}$ where the PDE residual is already low. We here call this procedure residual based adaptive exchange (RAE) and give the details in Supplementary Section S5.2 online. We note that similar refinement techniques have recently also been proposed and studied extensively in^51^ and^52^.

The dotted red line in Fig. 5 demonstrates that in our setting both methods yield similarly low residuals *r*(*t*) without overfitting the data. Since in RAE the number of PDE points stays the same during training, the computing time is the same as without RAR. In Fig. 5b it can be seen how both RAR and RAE add more PDE points around the timepoints where data is available.

We estimate the apparent diffusion coefficient *D* by averaging over 5 trainings with either RAR or RAE and learning rate decay from $10^{-3}$ to $10^{-4}$ or $10^{-4}$ to $10^{-5}$. The results are displayed in Fig. 6b together with the $\ell ^1$-norm (2) after training. It can be seen that for the same learning rate, both RAR and RAE yield similar results. A lower learning rate, however, leads to lower PDE residual and an estimated diffusion coefficient which is closer to the value 0.72 $\hbox {mm}^2\,\hbox {h}^{-1}$ from Valnes et al.^28^.

### Testing different patients

In Valnes et al.^28^, the same methodology was applied to two more patients, named ’REF’ and ’NPH2’. We here test how well the optimal hyperparameter settings found in section “MRI data” generalize to these patients. A similar subregion of the white matter is used but the voxels on the boundary of the domain were removed.

A PINN is trained with the following hyperparameters from section “MRI data” that yielded the lowest PDE residual after training: The number of minibatches is set to 20, training for $$10^5$$ epochs with ADAM and exponential learning rate decay from $$10^{-4}$$ to $$10^{-5}$$, and $$p=1$$ with RAR at $$1\times 10^4,\, 2\times 10^4, \ldots ,\, 9\times 10^4$$ epochs. The network architecture remains the same. For patient ’NPH2’ we find $$D=0.48$$ $$\hbox {mm}^2\,\hbox {h}^{-1}$$ while the FEM approach^28^ yields $$D=0.50$$ $$\hbox {mm}^2\,\hbox {h}^{-1}$$. We find $$D=0.41 $$ $$\hbox {mm}^2\,\hbox {h}^{-1}$$ for patient ’REF’ while the FEM approach^28^ yields $$D=0.50$$ $$\hbox {mm}^2\,\hbox {h}^{-1}$$.

## Discussion

We have tested both PINNs and FEM for assessing the apparent diffusion coefficient in a geometrically complex domain, a subregion of the white matter of the human brain, based on a few snapshots of T1-weighted contrast enhanced MR images over the course of 2 days. Both methodologies yield similar estimates when properly set up, that is; we find that the ADC is in the range (0.6–0.7) $\hbox {mm}^2\,\hbox {h}^{-1}$, depending on the method, whereas the DTI estimate is 0.4 $\hbox {mm}^2\,\hbox {h}^{-1}$. As such the conclusion is similar to that of Valnes et al.^28^. With a proper hyperparameter set-up, PINNs are as accurate as FEM and, given our implementation with GPU acceleration, roughly twice as fast as our current FEM implementation on MRI data as shown in Supplementary Section S4.1 online.

However, choosing such a set-up, i.e., hyperparameter setting, loss function formulation and training procedure, is still a priory not known and challenging. An automated way to find a suitable setting is needed. To this end automated approaches such as AutoML^53^ or Meta learning^54^, could be applied in the future. Moreover, theoretical guarantees are required, especially in sensitive human-health related applications.

Our results are in line with the frequent observation that the PDE loss weight is an important hyperparameter. Several works have put forth methodologies to choose the weights adaptively during training^8–11^, but in practice they have also been chosen via trial-and-error^43,55,56^. However, in settings with noisy data, it can not be expected that both data loss and PDE loss become zero. The ratio between PDE loss weight and data loss weight reflects to some degree the amount of trust one has in the data and the physical modeling assumptions, i.e., the PDE. In this work, we have made the modeling assumption that the data is governed by a diffusion equation, and hence require the PDE to be fulfilled. This provides a criterion for choosing a Pareto-optimal solution if the PINN loss is considered from a multi-objective perspective^57^.

From the mathematical point of view, we have sought the solution of a challenging nonlinear ill-posed inverse problem with limited and noisy data in both space and time. There can thus be more than one local minimum and the estimated solutions depend on the regularization and/or hyperparameters. Here, our main observation is that the diffusion coefficient recovered by PINNs approaches the FEM result when the hyperparameters are chosen to ensure that the PDE residual after training is sufficiently small.

In general, we think that the current problem serves as a challenging test case and is well suited for comparing PINNs and FEM based methods. Further, since the finite element approach is well-established and theoretically founded it can serve to benchmark PINNs. Our numerical results indicate that the norm of the PDE residual of the trained PINN correlates with the quality of the recovered parameter. This relates back to the finite element approach where the PDE residual is small since the PDE is explicitly solved. In our example, we have found that in particular two methodological choices help to significantly lower the PDE-residual in the PINNs approach: $\ell ^1$-penalization of the PDE and adaptive refinement of residual points.

From the physiological point of view, there are several ways to improve upon our modeling assumption of a diffusion equation with spatially constant, scalar diffusion coefficient. The microscopic bulk flow proposed by the glymphatic theory may, on the macroscopic scale, be mathematically modelled in the form of convection^40^, dispersion^58^, clearance^59^.

For instance, an estimate of the local CSF velocity can be obtained by the optimal mass transport technique^60^. From an implementational point of view, such methods fit well within our current framework since the PINN formulation is comparably easy to implement and the PDE does not have to be solved explicitly.

## Methods

### Approvals and MRI acquisition

The approval for MRI observations was retrieved by the Regional Committee for Medical and Health Research Ethics (REK) of Health Region South-East, Norway (2015/96) and the Institutional Review Board of Oslo University Hospital (2015/1868) and the National Medicines Agency (15/04932-7). The study participants were included after written and oral informed consent. All methods were performed in accordance with the relevant guidelines and regulations. Details on MRI data acquisition and generation of synthetic data can be found in the Supplementary Section S1 online.

### The PINN approach

In PINNs, our parameter identification problem can be formulated as an unconstrained non-convex optimization problem over the network parameters $\theta$ and the diffusion coefficient *D* as

4 $$\begin{aligned} \min _{\theta , D} {\mathcal {J}} + w_r {\mathcal {L}}_r, \end{aligned}$$

where $w_r > 0$ is a weighting factor. We model the concentration measurements by a fully connected neural network $c(x, t; \theta )$ where $x\in {\mathbb {R}}^3$ are spatial inputs and $t\in {\mathbb {R}}$ is the time input. The data loss ${\mathcal {J}}$ is defined as

5 $$\begin{aligned} {\mathcal {J}} = \frac{1}{N_d} \sum _{t_i \in {\mathcal {T}}} \sum _{x \in \Omega _d} (c(x, t_i; \theta ) - c^d(x, t_i))^2, \end{aligned}$$

where $\Omega _d$ is a discrete finite subset of $\Omega$, ${\mathcal {T}}=\{0,7,24,46\}$ h, and $N_d$ denotes the number of space-time points in ${\mathcal {T}} \times \Omega$ where we have observations. The PDE loss term ${\mathcal {L}}_{r}$ is defined as

6 $$\begin{aligned} {\mathcal {L}}_{r} = \frac{1}{|{\mathcal {P}}|}\sum \limits _{(x, t) \in {\mathcal {P}}} |\partial _t c(x,t; \theta ) - D \Delta c(x, t; \theta )|^p, \end{aligned}$$

where $p \in [1, \infty )$, the set ${\mathcal {P}}$ consists of $N_r$ points in $\tau \times \Omega _r$, $\tau \subset [0,T]$, and $\Omega _r \subset \Omega$ is a set of $N_p=|{\mathcal {P}}|$ coordinates $x \in {\mathbb {R}}^3$ that lie in the *interior* of the domain $\Omega$. The sampling strategy to generate ${\mathcal {P}}$ is explained in detail in Supplementary Section S3 online. In this work we test training with both $p=2$ and $p=1$. It is worth noting that boundary conditions are not included (in fact, they are often not required for inverse problems^3^) in the PINN loss function (4), allowing us to sidestep making additional assumptions on the unknown boundary condition. The initial condition is taken to be the first image at $t=0$ and simply enters via the data loss term (5). A detailed description of the network architecture and other hyperparameter settings can be found in Supplementary Section S3 online.

### The finite element approach

Our parameter identification problem describes a nonlinear ill-posed inverse problem^61–63^. As a comparison baseline for the PINN approach, we build on the numerical realization of Valnes et al.^28^ and define the PDE constrained optimization problem^64^ as

7 $$\begin{aligned} \min \limits _{D, g} \sum _{t_i \in {\mathcal {T}}} \int _\Omega (c(x, t_i; D,g) - c^d(x, t_i))^2 \, \mathrm {d}x + \frac{1}{2} \int _0^T \int _{\partial \Omega } \left( \alpha |g|^2 + \beta |\frac{\partial }{\partial t} g|^2 + \gamma | \nabla g |^2 \right) \mathrm {d}S \mathrm {d} t, \end{aligned}$$

where, similar to^28^, the second term is Tikhonov regularization with regularization parameters $\alpha , \beta , \gamma > 0$ and $c=c(x,t, D, g)$ solves (1) with boundary and initial conditions

8 $$\begin{aligned} c(x,t)&= g(x,t) \quad \text {on } \partial \Omega \times (0, T), \end{aligned}$$

9 $$\begin{aligned} c(x, 0)&= 0 \quad \text {in } \Omega . \end{aligned}$$

To determine *c* for given (*D*, *g*), the partial differential equation is considered in a weak variational form and discretized in time, by using a finite difference method, and in space, by using finite elements. This leads to a sequence of linear systems of equations, which needs to be solved to obtain the state *c*. Hence, in the finite element approach, the state, that is used to evaluate the objective function, fulfills the weak form of the partial differential equation in a discretized sense. In order to compute the derivative of the functional (7) with respect to the controls (*D*, *g*), automated differentiation techniques are applied in a similar fashion as backpropagation is applied for neural networks. A detailed description of the mathematical and implementation details can be found in Supplementary Section S3 online.

### Parameterization of the diffusion coefficient

Previous findings^35,40,59,60^ indicate that diffusion contributes at least to some degree to the distribution of tracers in the brain. It can thus be assumed that a vanishing diffusion coefficient is unphysical. This assumption can be incorporated into the model by parameterizing *D* in terms of a trainable parameter $\delta$ as

10 $$\begin{aligned} D(\delta ) = D_{\mathrm {min}} + \sigma (\delta ) D_{\mathrm {max}}, \end{aligned}$$

where $\sigma (x) = (1+\exp (-x))^{-1}$ denotes the logistic sigmoid function. In all results reported here, we initialize with $\delta = 0$ and set $D_{\mathrm {min}} = 0.1 \, \text {mm}^2 \, \text {h}^{-1}$ and $D_{\mathrm {max}} = 1.2 \, \text {mm}^2\, \text {h}^{-1}$. This parameterization with a sigmoid function effectively leads to vanishing gradients $|\frac{\partial D}{\partial \delta }|$ for $|\delta | \gg 1$. In section “Synthetic data with noise” we demonstrate that this choice of parameterization can help to avoid instabilities that occur during PINN training without parameterization, i.e.

11 $$\begin{aligned} D = \delta . \end{aligned}$$

The reason to introduce a $D_{\mathrm {min}} > 0$ is to avoid convergence into a bad local minimum. For the finite element approach, we did not observe convergence into a local minimum where $D=0$, and hence used the parameterization (11).

## Supplementary Information

Supplementary Information.

## Data Availability

The datasets analyzed in the current study are available from the corresponding author upon request.
