# Supplementary material for: Investigating molecular transport in the human brain from MRI with physics-informed neural networks
Source: Sci Rep. 2022 Sep 14;12:15475. doi: 10.1038/s41598-022-19157-w (PMC9474534; doi:10.1038/s41598-022-19157-w)
Supplement: Supplementary file 1 — Supplementary Information. [file 41598_2022_19157_MOESM1_ESM.pdf]

Supplementary information for ’Investigating molecular transport in the human brain from MRI with physics-informed neural networks’

Bastian Zapf[1], Johannes Haubner[2], Miroslav Kuchta[2], Geir Ringstad[3,4], Per Kristian Eide[5,6], and Kent-Andre Mardal[1,2,*]

[1]University of Oslo, Faculty of Mathematics and Natural Sciences, Oslo, 0851, Norway
[2]Simula Research Laboratory, Department of Numerical Analysis and Scientific Computing, Oslo, 0164, Norway
[3]Oslo University Hospital, Department of Radiology, Oslo, 0372, Norway
[4]Sorlandet Hospital, Department of Geriatrics and Internal medicine, Arendal, 4838, Norway
[5]Oslo University Hospital, Department of Neurosurgery, Oslo, 0372, Norway
[6]University of Oslo, Institute of Clinical Medicine, Oslo, 0372, Norway
[*]kent-and@simula.no

S1 Data Generation

S1.1 MRI Data

The data under consideration in this study is based on MRI scans taken of a patient who was imaged at Oslo University Hospital in Oslo, Norway. The patient was diagnosed with normal pressure hydrocephalus and is referred to as "NPH1" in[1].

The imaging protocol starts with the acquisition of a baseline MRI before 0.5 mL of a contrast agent (1 mmol/mL gadobutrol) is injected into the CSF at the spinal canal (intrathecal injection). The pulsating movement of CSF transports the tracer towards the head where it enters the brain. The contrast agent alters the magnetic properties of tissue and fluid, and in subsequently taken MRI, enriched brain regions display changes in MR signal relative to the baseline MRI. From the change in signal we estimate the concentration of tracer per voxel at timepoints 0, 7, 24 and 46 hours after injection. Further details on the MRI acquisition and tracer concentration estimation can be found in[1].

We next use FreeSurfer[2] to segment the baseline image into anatomical regions and obtain binary masks for white and gray matter. The human brain has many folds and represents a highly complex geometry. To limit the intrinsically high computing requirements of inverse problems, we focus on a subregion of the white matter shown in main Fig. 2a.

In the following, we describe how this data is processed further to obtain patient-specific finite element meshes to generate synthetic test data by simulation.

S1.2 Synthetic data

We use the surface meshes created during the brain segmentation with FreeSurfer[2] to create finite element meshes of the full brain. In detail, the first step in the mesh generation pipeline is to load Freesurfer brain surfaces into SVMTK[3], a Python library based on CGAL[4], for semi-automated removal of defects and creation of high quality finite element meshes. Details on SVMTK and the mesh generation procedure can be found in[3].

Using FEM we then solve the PDE (1) with boundary and initial conditions (8), (9) with a diffusion coefficient $D_0 = 0.36\,\mathrm{mm}^2\,\mathrm{h}^{-1}$ for the domain Ω being the whole brain. This value for the diffusion coefficient of gadubutrol was estimated in[1] from diffusion tensor imaging (DTI). In detail, we discretize (1) using the Crank-Nicolson scheme and use integration by parts to transform (1) into a variational problem that is solved in FEniCS[5] with continuous linear Lagrange elements. We use a high resolution mesh with 3×10^5 vertices (10^6 cells) and small time step of 16 min. In combination with the Crank-Nicolson scheme, this minimizes effects of numerical diffusion. For the initial condition (9) we assume no tracer inside the brain at $t = 0$, i.e. $c_0 = 0$. The boundary condition (8) is assumed to be spatially homogeneous while we let it vary in time as

$$g(t) = \begin{cases} 2t/T & \text{for } 0 \leq t \leq T/2 \\ 2 - 2t/T & \text{for } T/2 \leq t \leq T. \end{cases} \tag{S1}$$

This choice leads to enrichment of tissue similar to what is observed experimentally over the timespan of $T = 46$ hours. Finally, we interpolate the finite element solution $c(x,t)$ between mesh vertices and evaluate it at the center coordinates x_{ijk} of the voxels ijk inside the region of interest Ω and store the resulting concentration arrays c_{ijk} at 0, 7, 24 and 46 hours. With this downsampling procedure, we are then able to test the methods within the same temporal and spatial resolution as available from MRI. Finally, we remove all the voxels that are not within our white matter subdomain of interest Ω.

S1.3 Synthetic data with artifical noise

We test the susceptibility of the methods with respect to noise by adding to the data perturbations drawn randomly from the normal distribution $\mathcal{N}(0, \sigma^2)$. We refer to the standard deviation σ as noise level hereafter. Since negative values for the concentration c are nonphysical, we threshold negative values to 0, i.e. the noise-corrupted voxel values are computed as

$$c_{ijk} = \max\{0, c_{ijk} + \eta\} \quad \text{where} \quad \eta \sim \mathcal{N}(0, \sigma^2). \tag{S2}$$

In all the results presented in this work, we choose $\sigma = 0.05$. This corresponds to 5 % of the maximum value of $c = 1$ in the simulated measurements c_{ijk} and allows to reproduce some of the characteristic difficulties occurring when applying the PINN to the clinical data considered here.

S2 Assessing the validity of assuming a constant diffusion coefficient

We here provide arguments to motivate our modeling assumption of a scalar diffusion coefficient that is spatially constant in the region of interest Ω.

S2.1 Using tensor valued spatially varying diffusivity has little influence on forward simulations of tracer

In this section we assess the sensitivity of simulated tracer concentration with respect to two different modeling choice for the diffusivity D.

To this end, we use the diffusion tensor image (DTI) available for the patient under consideration in this work and use it to estimate the spatially varying diffusion tensor $\mathsf{D}(x) \in \mathbb{R}^{3\times 3}$ for the gadobutrol tracer as described in[1]. An illustration of the mean apparent diffusion coefficient $\frac{1}{3}\mathrm{tr}\mathsf{D}(x)$ can be found in Fig. S1d.

We now use this diffusion tensor to perform forward simulations of tracer spreading into the patient's brain using FEM as described in Section S1.2. In detail, instead of solving the scalar diffusion equation (1) we solve

$$\partial_t c(x,t) = \nabla \cdot (\mathsf{D}(x)\nabla c(x,t)) \quad \text{for} \quad (x,t) \in \Omega \times (0,T) \tag{S3}$$

$$c(x,0) = 0 \quad \text{for} \quad x \in \Omega \tag{S4}$$

$$c(x,t) = g(x,t) \quad \text{for} \quad (x,t) \in \partial\Omega \times (0,T) \tag{S5}$$

for the two distinct choices of $\mathsf{D}(x)$ stated below. Here, the domain Ω is set to be the whole brain. The initial condition is zero as in Section S1.3, but here we use a linear interpolation of data as boundary condition g. In detail, we define the boundary condition as

$$g^d(x,t) = c_i^d(x) + (c_{i+1}^d(x) - c_i^d(x))\frac{t - t_i}{t_{i+1} - t_i} \quad \text{for} \quad t_i \leq t \leq t_{i+1} \tag{S6}$$

where $t_i = \{0, 6, 24, 46\}\,\mathrm{h}$ are the observation times and c_i^d is the tracer estimate from MRI at time t_i.

Model 1: Tensor valued diffusivity. Here, we directly use the spatially varying diffusion tensor

$$\mathsf{D}(x) \in \mathbb{R}^{3\times 3} \tag{S7}$$

for gadobutrol as obtained from DTI to solve PDE (1).

Model 2: Constant scalar diffusivity in white and gray matter. For this model, we compute regional mean apparent diffusion coefficients

$$\overline{D} = \frac{1}{3}\frac{1}{|\Omega|}\int_\Omega \mathrm{tr}\mathsf{D}(x)\,\mathrm{d}x. \tag{S8}$$

We consider two regions, white and gray matter, and obtain

$$\overline{D}_w = 0.42 \pm 0.19\,\mathrm{mm^2h^{-1}} \quad \overline{D}_g = 0.46 \pm 0.21\,\mathrm{mm^2h^{-1}}. \tag{S9}$$

The PDE (S5) is then solved with

$$\mathsf{D}(x) = D(x)\mathbb{1}; \quad D(x) = \begin{cases} \overline{D}_g & \text{for } x \in \text{ gray matter} \\ \overline{D}_w & \text{for } x \in \text{ white matter} \end{cases} \tag{S10}$$

where $\mathbb{1}$ denotes the identity matrix.

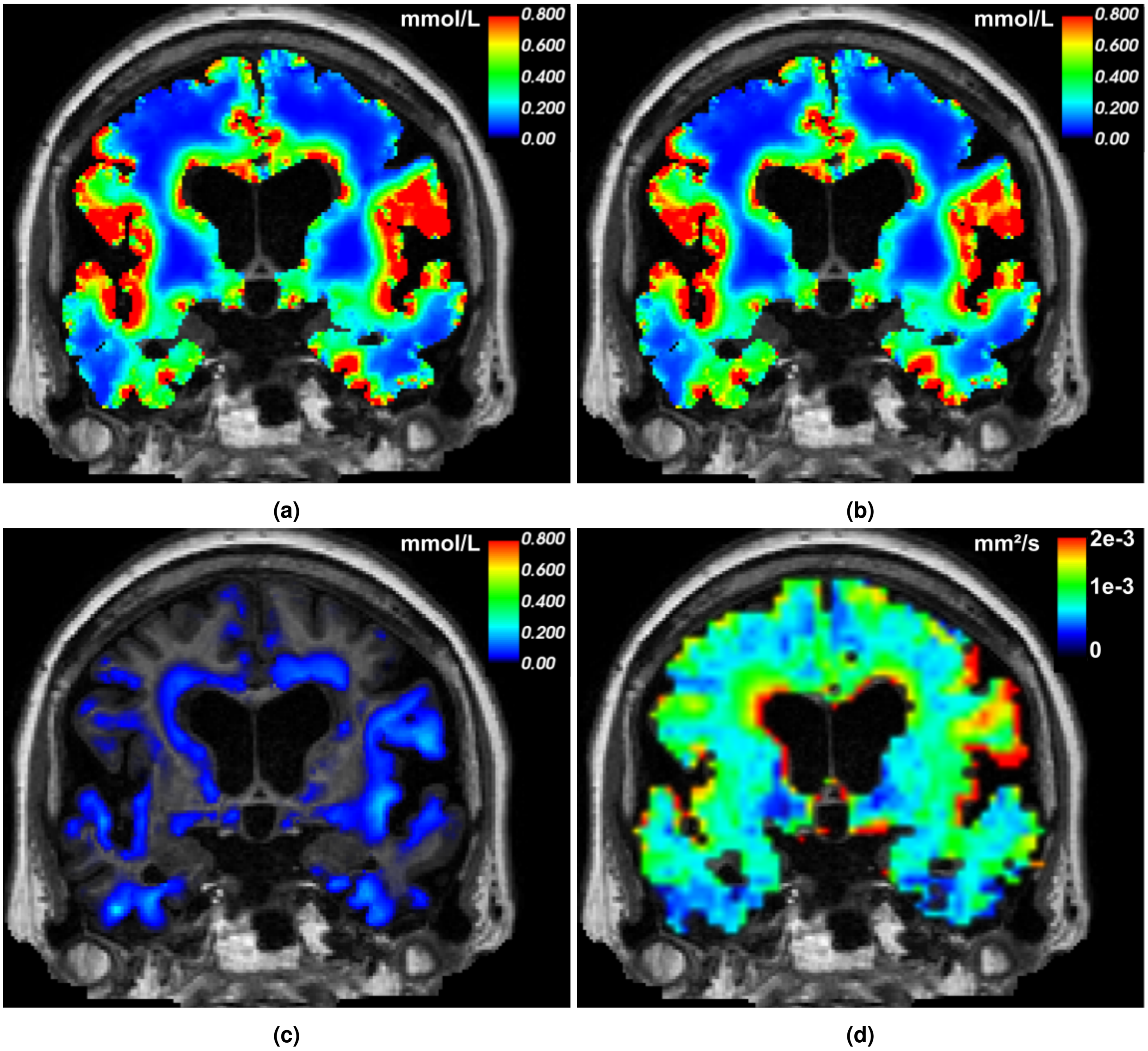

Figure S1. (a) Simulated tracer after 24 h obtained with DTI based Model 1 (S7). (b) Simulated tracer after 24 h obtained with scalar diffusion Model 2 (S10). The difference between (a) and (b) is barely visible to the eye, and the absolute difference is shown in (c) using the same scale. (d) the mean diffusivity $\frac{1}{3}\mathrm{trD}$.

In Fig. S1a and S1b we exemplarily display visualizations of the simulated tracer after 24 h (where the amount of tracer reaches its maximum in clinical observations) for both models. The difference is barely visible to the eye, and the relative ℓ^1-error between the tracer at 24 h simulated with the two models is only 8.8 %. Hence, it can not be expected that in an inverse setting with three snapshots of real data one can reliably differentiate by which of these two diffusion models the tracer distribution observed with MRI is governed.

S2.2 DTI characteristics suggest nearly isotropic diffusion

We next present some details on the diffusion tensor image available for the patient under consideration in this work. Figure S2a illustrates the fractional anisotropy

$$FA = \frac{3}{2}\frac{(\lambda_1 - MD)^2 + (\lambda_2 - MD)^2 + (\lambda_3 - MD)^2}{\lambda_1^2 + \lambda_2^2 + \lambda_3^2} \in [0,1] \tag{S11}$$

where

$$MD = \frac{1}{3}\mathrm{trD} \tag{S12}$$

is the mean diffusivity and λ_i, $i = 1, 2, 3$ are the eigenvalues of the diffusion tensor. It can be seen that the FA is rather small in most of the brain, in fact the mean $FA \approx 0.25$. The minimum value $FA = 0$ corresponds to isotropic diffusion, while $FA = 1$ corresponds to diffusion restricted to only one direction. Hence the value $FA \approx 0.25$ is in line with our assumption of a scalar, constant diffusion coefficient. Figure S2b further illustrates the distribution of eigenvalues in our region of interest. It can be seen that the ratio between largest and smallest eigenvalue is roughly 1.8, further justifying our modeling assumption of isotropic diffusion.

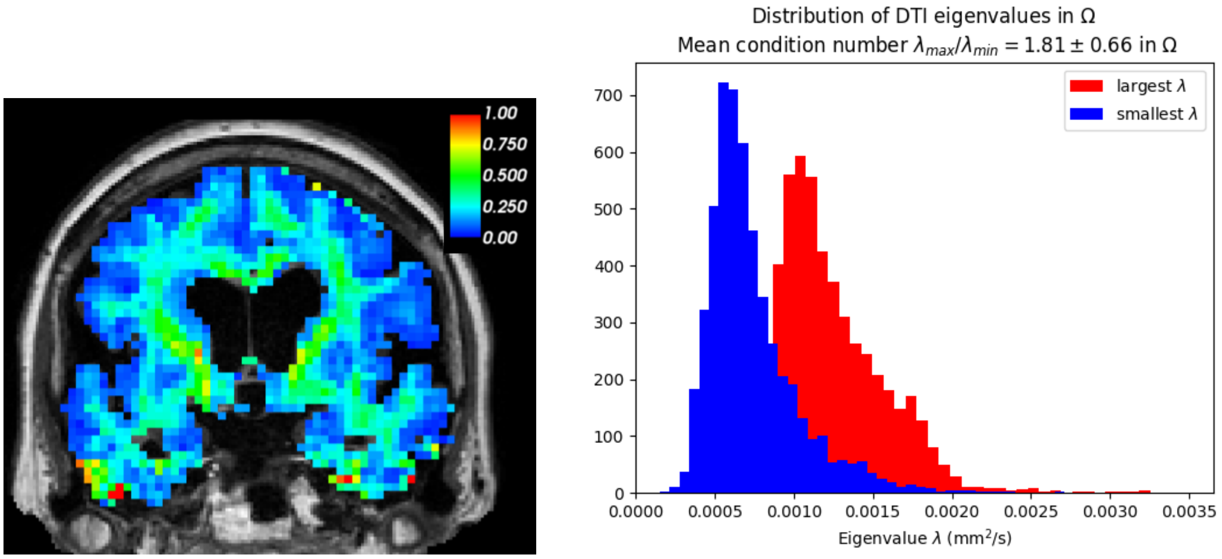

(a) **(b)**

Figure S2. (a) Fractional anisotropy and (b) distribution of smallest and largest eigenvalues in our domain of interest Ω for the diffusion tensor image.

S3 Hyperparameter settings

In our PINN approach, we model $c : (x,t) \to \mathbb{R}, x \in \mathbb{R}^3,\ t \in [0,T]$ by a feedforward neural network with 9 hidden layers and 64 neurons in each layer and hyperbolic tangent as activation function together with Glorot initialization[6] for all results presented here. We have also experimented with larger networks, different and adaptive activation functions but have not observed significant differences for a range of choices in terms of convergence rates or accuracy. We note that since the raw data is already a 3-D array representing grid structured data, using (physics-informed) convolutional neural networks instead of fully connected networks might yield benefits such as training speed up. In this work, however, we decide to focus on tuning the loss function formulation and find that using a fully connected network in combination with a properly tuned loss function yields results that are consistent with the FEM approach.

The network furthermore has an input normalization layer with fixed parameters to normalize the inputs to the range $[-1,1]$. To set these weights, we first compute the smallest bounding box containing all points $x = (x_1,x_2,x_3) \in \Omega$ to obtain lower and upper bounds l_i, u_i, $i = 1,2,3$ such that $l_i \le x_i \le u_i$ for all $x \in \Omega$. The first layer normalizes the inputs as

$$t \hookleftarrow 2\frac{t}{T} - 1,\ x_i \hookleftarrow 2\frac{x_i - l_i}{u_i - l_i} - 1 \tag{S13}$$

for $i = 1,2,3$ and with $T = 46\,\mathrm{h}$ (last MRI acquisition timepoint).

If not stated otherwise, we use $N_p = 10^6$ space-time points (x_1,x_2,x_3,t) for the evaluation of the PDE loss (4). We found that this number was either sufficiently high to reach accurate recovery of the diffusion coefficient, or more sophisticated refinement techniques like residual-based adaptive refinement (RAR)[7] were needed instead of simply using more PDE points. The samples for the spatial coordinates x_1,x_2,x_3 are generated by first drawing a random voxel i inside Ω. The voxel center coordinates (x_1^i, x_2^i, x_3^i) are then perturbed to obtain $x_1 = x_1^i + dx$ where x_1^i is the x_1-coordinate of the center of a randomly drawn voxel i, and similarly for x_2 and x_3. The perturbation dx is drawn from the uniform distribution $\mathcal{U}([-0.5\,\mathrm{mm}, 0.5\,\mathrm{mm}])$ and ensures that (x_1,x_2,x_3) lies within the voxel i (the voxels correspond to a volume of $1\,\mathrm{mm}^3$). The values for t are chosen from a latin hypercube sampling strategy over the interval $[0,T]$. We furthermore normalize the input data c^d by the maximum value such that $0 \le c^d \le 1$. In both the simulation dataset and the MRI data considered here, we use four images and the same domain Ω. The binary masks describing Ω consist of roughly 0.75×10^4 voxels, i.e., the four images (at 0, 7, 24 and 46 hours) yield a total of $N_d = 3 \times 10^5$ data points. Due to the large number of data and PDE points, we use minibatch sampling of the PINN loss function (4) and minimize it using the ADAM optimizer[8]. The learning rate η as well as potential learning rate decay schemes are an important hyperparameter, and we specify the used values in each section. The training set is divided into 20 batches, corresponding to 10^4 and 5×10^4 samples per minibatch in the data and PDE loss term, respectively.

Details on the implementation of the minibatch sampling strategy are presented in Section S5. It is worth noting here that our main reason to use minibatch sampling are not memory limitations. The graphics processing units (NVIDIA A100-SXM4) that we use to train the PINN have 80 GB of memory. This is enough to minimize the PINN loss function (4) with $N_d = 3 \times 10^5$ and $N_p = 10^6$ data and PDE loss points in a single batch. Our reason to use minibatch sampling is that the stochasticity of minibatch gradient descent helps to avoid local minima, see, e.g., Chapter 8 in[9]. In Section S4 below we perform a systematic study using different minibatch sizes and find that smaller batch sizes are preferable in our setting since they yield more accurate recovery of the diffusion coefficient (for a fixed number of epochs).

As for the finite element approach, we discretize (1) in time using the Crank-Nicolson scheme and 48 time steps. We then formulate the PDE problem as variational problem and solve it in FEniCS[5] using the finite element method. To limit the compute times required, we use a time step size of 1 h and continuous linear Lagrange elements. We further use linear interpolation of the data as a starting guess for the boundary control g,

$$g(x,t) = c^d(x,t_i) + \frac{c^d(x,t_{i+1}) - c^d(x,t_i)}{t_{i+1} - t_i}(t - t_i) \tag{S14}$$

for $t_i \in \mathcal{T} = \{0,7,24,46\}\,\mathrm{h}$ and $t_i \le t \le t_{i+1}$. We then use dolfin-adjoint[10] to compute gradients of the functional (7) with respect to D and g and optimize using the L-BFGS method.

In terms of degrees of freedom (optimization parameters), these settings result in 33665 weights in the neural network. For the finite element approach, the degrees of freedom depend on the number of vertices on the boundary of the mesh since the control is the boundary condition g. Our mesh for Ω has 33398 cells on the boundary. For 48 time steps, this yields $48 \times 33398 = 1.6 \times 10^6$ degrees of freedom.

S4 Validation on synthetic data

We verify the implementation of the two approaches by considering synthetic data without noise, cf. Fig. 2b, in the white matter subregion Ω depicted in Fig. 2a.

Table S1. Iterations needed for convergence with both approaches on the different data sets tested in this study. Numbers in brackets indicate typical values for the compute times.

	synthetic with noise	MRI data
FEM	1,000 (45-50 h)	1,000 (45-50 h)
PINN	20,000 (3 h)	100,000 (20 h for RAR, 15 h for RAE)

For the PINN approach we test different minibatch sizes for three different optimization schemes using the ADAM optimizer: (i) fixed learning rate 10^{-3} and $p = 2$, (ii) fixed learning rate 10^{-3} while we switch from $p = 2$ to $p = 1$ after half the epochs and (iii) using initial learning rate 10^{-3} that decays exponentially during training to 10^{-4} and $p = 2$. Table S2 tabulates the relative error between the learned diffusion coefficient and the ground truth D_0 for a wide range of parameters. We find that (a) in general smaller batch sizes result in more accurate results and (b) the results are both most stable and accurate when using exponentially decaying learning rate. Notably, the PINN recovers the ground truth diffusion coefficient D_0 to up to 1 % accuracy when using the learning rate decay optimization scheme. These result are in line with[11] where increased accuracy in parameter recovery was observed for smaller batch sizes. However, there are also settings where full batch optimization with L-BFGS improves PINN performance in parameter identification problems[12].

For the finite element approach, Table S3 presents the accuracy of the recovered diffusion coefficient. According to the theoretical results, decreasing regularization parameters leads to higher accuracy but less well conditioned optimization problems. This is in line with the results presented in Table S3. The finite element approach with appropriate regularization parameters and the PINN approach yield comparably accurate results.

S4.1 Computational effort

We here list the computing times to estimate the diffusion coefficient with our implementation of the FEM and PINN approaches presented in the main text. It should be noted that neither of these implementations have been optimized to reduce the compute times, and that the times required for convergence vary between datasets.

Our implementation of the FEM approach using dolfin-adjoint[10] with a mesh containing 91849 cells and 23307 vertices (whereof 33398 and 16693 are on the boundary, respectively) requires around 45-48 hours computing time on noisy synthetic data for the 1,000 iterations until convergence as shown in Supplementary Fig. S3a using a single Intel Xeon Gold CPU. As for the PINN approach, on noisy synthetic data, convergence was achieved when terminating the optimization after 20,000epochs of ADAM with data and PDE batch sizes of 1.5×10^4 and 5×10^4, respectively. With our implementation in PyTorch[13] this takes about 3 hours on a NVIDIA A100-SXM4.

As for training on real MRI data, the FEM approach was usually converged after 1,000 iterations as well, while the PINN approach required lower learning rate and thus more epochs. The PINN results presented in the text were obtained after 100,000 epochs which usually was sufficient to converge. The compute time varies between sampling strategies (for PINNs) and workload on the cluster node for the particular simulation (for both approaches). For PINNs, the compute time was around 20 h for RAR and 15 h for RAE. The results are sumarized in S1.

We have also performed additional numerical experiments where we use residual based adaptive exchange and train with both ADAM and L-BFGS optimizer after every refinement. This strategy is similar to[14]. It emerges that a combination of low learning rates for ADAM, adaptive residual based refinement and ℓ^1 norm for the PDE loss allows us to successfully train with a combination of Adam + L-BFGS. The results after training with ADAM + L-BFGS are consistent with the ADAM results reported in the manuscript, while roughly half the optimization time is required.

S4.2 PINN solution of the synthetic testcase

Table S2. Average rel. error $|D_{\text{pinn}} - D_0|/D_0$ in % after 2×10^4 epochs training on synthetic data without noise, with Algorithm 1. We average over 5 runs, numbers in brackets are standard deviation.

Optimization scheme	n_r \ n_d	10^4	5×10^4	10^5
ADAM lr = 1e-3 $p = 2$	10000	2 (0)	2 (1)	3 (1)
	33334	4 (0)	12 (6)	9 (1)
	50000	7 (1)	5 (0)	2 (0)
	100000	7 (1)	55 (18)	59 (18)
	166667	8 (1)	24 (17)	50 (23)
	333334	8 (1)	38 (4)	50 (16)
ADAM lr = 1e-3 p=2 $\rightarrow$ p=1	10000	2 (0)	1 (0)	2 (0)
	33334	2 (0)	2 (0)	2 (0)
	50000	2 (1)	2 (0)	2 (1)
	100000	2 (1)	58 (27)	50 (25)
	166667	2 (1)	3 (2)	68 (4)
	333334	2 (0)	0 (0)	62 (6)
ADAM exp lr decay 1e-3 $\rightarrow$ 1e-4 $p = 2$	10000	1 (0)	1 (0)	1 (0)
	33334	1 (0)	1 (1)	1 (0)
	50000	1 (1)	1 (0)	1 (0)
	100000	1 (0)	10 (6)	72 (0)
	166667	1 (1)	4 (2)	23 (26)
	333334	1 (0)	4 (4)	59 (23)

S4.3 Finite element solution of the synthetic testcases

Table S3. Rel. error $|D - D_0|/D_0$ for the FEM approach (7), different regularization parameters, 3 measurement points, clean data, 1,000 iterations. Convergence of the optimization is demonstrated in Fig. S3a.

α	β \ γ	0.0	0.01	1.0
10^{-6}	0.001	9	8	261
	0.01	1	5	261
	0.1	11	10	261

Table S4. Rel. error $|D - D_0|/D_0$ in % for the finite element approach (7), different regularization parameters and parameterizations $D(\delta)$. Failure of the algorithm is indicated by the symbol "x". Convergence plots for the optimization are given in Fig. S3b.

Parameterization		$D = \delta$ (11)		$D = D(\delta)$ (10)	
α	β \ γ	0.0	0.01	0.0	0.01
10^{-6}	0.0	43	10	35	2
	0.01	8	x	16	8
	0.1	4	x	3	11
10^{-4}	0.0	44	x	6	20
	0.01	5	13	2	14
	0.1	6	12	5	12

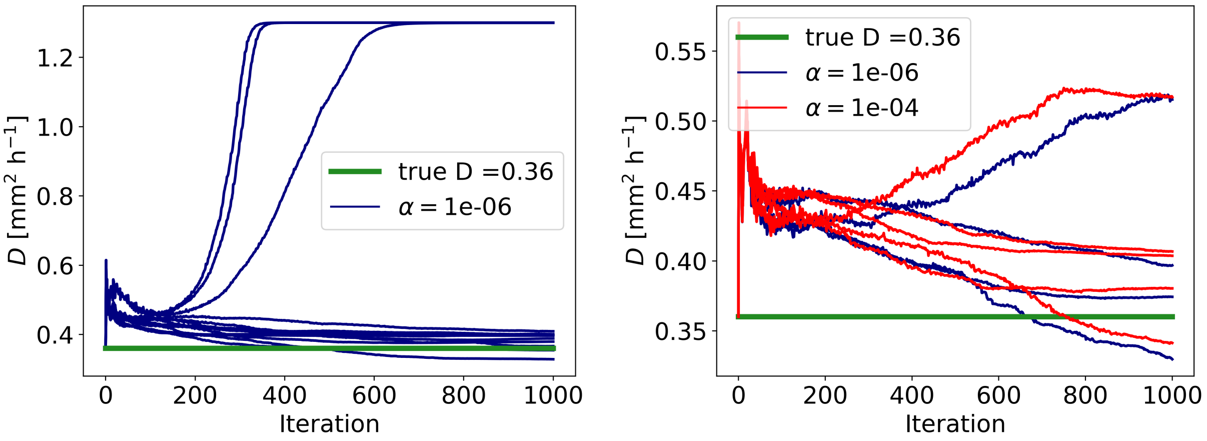

(a) **(b)**

Figure S3. Convergence plots for the FEM regularization parameters presented in Tables S3 (synthetic data) and S4 (synthetic data with noise). Different lines correspond to different combinations of regularization parameters $(\beta,\gamma) = \{0.001, 0.01, 0.1\} \times \{0, 0.01, 1\}$ and $\{0, 0.01, 0.1\} \times \{0, 0.01\}$ tested for synthetic and noisy synthetic data, respectively.

S5 Details on the PINN training procedures

S5.1 Minibatch sampling strategy

Algorithm 1 Minibatch training

Input: neural network c with parameters θ, data minibatch size n_d, PDE minibatch size n_r, RAR checkpoints $\{i_1, \dots, i_n\}$, epochs, learning rate η, initial guess D for the diffusion coefficient, input-data pairs $\{(\mathbf{x}_k^d, c_k^d) \in \mathbb{R}^4 \times \mathbb{R}\}_{1 \le k \le N_d}$, PDE space-time points $\mathcal{P} = \{\mathbf{x}_k \in \mathbb{R}^4\}_{1 \le k \le N_r}$

compute number of data batches b_d =ceil(N_d/n_d)
compute number of PDE batches b_r = ceil(N_r/n_r)
Set b =max(b_d, b_r)
for i in range(epochs) **do**
  if i $\in$ RAR checkpoints **then**
    add points to $\mathcal{P}$ with either procedure 2 or 3
  end if
  randomly split $\{(\mathbf{x}_k^d, c_k^d)\}$ into subsets $\mathcal{D}_{1 \le j \le b_d}$
  randomly split $\{\mathbf{x}_r^k\}$ into subsets $\mathcal{R}_{1 \le j \le b_r}$
  # Iterate over all minibatches
  for j in range(b) **do**
    # Start from beginning should you reach the last subset in $\mathcal{D}_{b_k}$ or $\mathcal{R}_{b_r}$, respectively (Happens if $b_r \neq b_d$):
    Set $j_d = j \bmod b_d$, $j_r = j \bmod b_r$
    # Compute losses on subsets
    $\mathcal{L} = \frac{1}{|\mathcal{R}_{j_d}|} \sum_{\mathbf{x}^d, c^d \in \mathcal{D}_{j_d}} \left(c(\mathbf{x}^d) - c^d\right)^2$
    $\mathcal{L}$ += $\frac{1}{|\mathcal{R}_{j_r}|} \sum_{\mathbf{x} \in \mathcal{R}_{j_r}} |\partial_t c(\mathbf{x}) - D\Delta c(\mathbf{x})|^p$
    # update parameters θ
    θ -= $\eta \nabla_\theta \mathcal{L}$
    # update diffusion coefficient D
    D -= $\eta \nabla_D \mathcal{L}$
  end for
end for

S5.2 Residual based refinement

Algorithm 2 Refinement step with the RAR procedure as in "Procedure 2.2" in[7] adapted to the nomenclature in our work.

Input: The set of N_r PDE points $\mathcal{P}$, PDE residual $r(x,t)$, number m of points to add per refinement step, number n of points to test the residual
Output: refined set of PDE points $\mathcal{P}$
1: Compute the absolute value of the PDE residual $|r(x,t)|$ at n random samples $\mathcal{S} = \{(x_1,t_1),\dots,(x_n,t_n)\}$ from $\Omega_r \times \tau$
2: Sort $\mathcal{S}$ by decreasing residual $|r(x,t)|$ and keep only the first m points in $\mathcal{S}_m$
3: **return** The refined set of $N_r + m$ points $\mathcal{P} \cup \mathcal{S}_m$

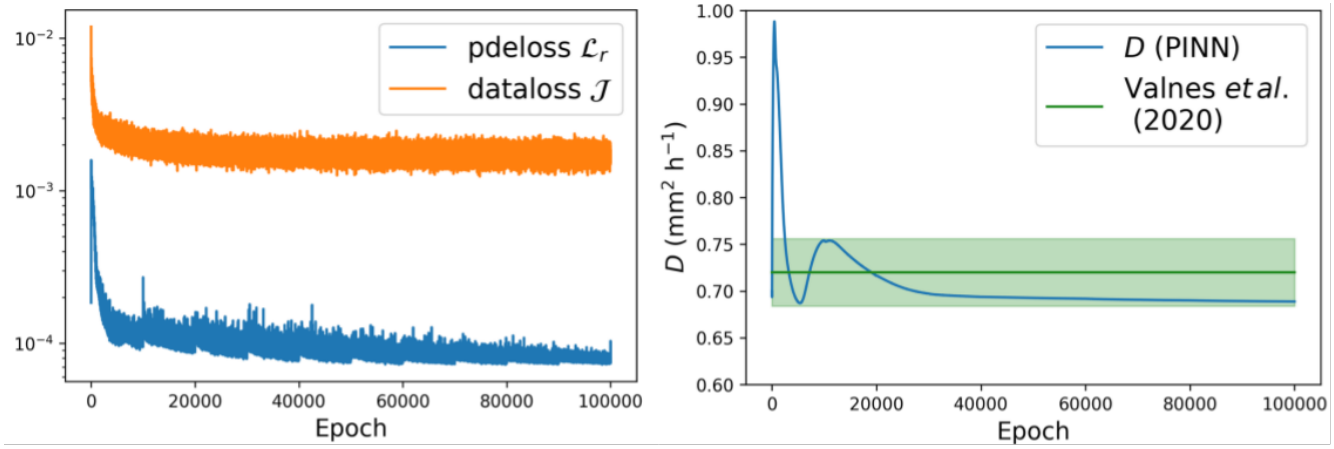

Figure S4. (Left) Convergence plots for the PINN losses trained with RAR on MRI data. (Right) Diffusion coefficient during PINN training. Exponential learning rate decay from 10^{-4} to 10^{-5} with RAR and $p = 1$.

Algorithm 3 Refinement step with the RAE procedure, a modification of the RAR procedure as described under "Procedure 2.2" in[7].

Input: The set of N_r PDE points $\mathcal{P}$, PDE residual $r(x,t)$, number m of points to add per refinement step, number n of points to test the residual
Output: refined PDE points $\mathcal{P}$
1: Compute the absolute value of the PDE residual $|r(x,t)|$ at n random samples $\mathcal{S} = \{(x_1,t_1),\dots,(x_n,t_n)\}$ from $\Omega_P \times \tau$
2: Sort $\mathcal{S}$ by decreasing residual $|r(x,t)|$ and keep only the first m points in $\mathcal{S}_m$
3: Compute the PDE residual $|r(x,t)|$ at the points in $\mathcal{R}$
4: Sort $\mathcal{R}$ by increasing residual $|r_{\mathcal{R}}(x,t)|$ and keep only the first $N_r - m$ points in $\mathcal{R}_{N_r - m}$
5: **return** The set of N_r refined points $\mathcal{R}_{N_r - m} \cup \mathcal{S}_m$

References

1. Valnes, L. M. *et al.* Apparent diffusion coefficient estimates based on 24 hours tracer movement support glymphatic transport in human cerebral cortex. *Sci. Reports* **10**, 9176, DOI: 10.1038/s41598-020-66042-5 (2020). Number: 1 Publisher: Nature Publishing Group.
2. Fischl, B. FreeSurfer. *NeuroImage* **62**, 774–781, DOI: 10.1016/j.neuroimage.2012.01.021 (2012).
3. Mardal, K.-A., Rognes, M. E., Thompson, T. B. & Valnes, L. M. Simulating Anisotropic Diffusion in Heterogeneous Brain Regions. In Mardal, K.-A., Rognes, M. E., Thompson, T. B. & Valnes, L. M. (eds.) *Mathematical Modeling of the Human Brain: From Magnetic Resonance Images to Finite Element Simulation*, Simula SpringerBriefs on Computing, 97–107, DOI: 10.1007/978-3-030-95136-8_6 (Springer International Publishing, Cham, 2022).
4. Project, T. C. *{CGAL} User and Reference Manual* (CGAL Editorial Board, 2022), 5.4 edn.

5. Alnæs, M. *et al.* The fenics project version 1.5. *Arch. Numer. Softw.* **3** (2015).

6. Glorot, X. & Bengio, Y. Understanding the difficulty of training deep feedforward neural networks. In *Proceedings of the Thirteenth International Conference on Artificial Intelligence and Statistics*, 249–256 (JMLR Workshop and Conference Proceedings, 2010). ISSN: 1938-7228.

7. Lu, L., Meng, X., Mao, Z. & Karniadakis, G. E. DeepXDE: A deep learning library for solving differential equations. *SIAM Rev.* **63**, 208–228, DOI: 10.1137/19M1274067 (2021). Publisher: Society for Industrial and Applied Mathematics.

8. Kingma, D. P. & Ba, J. Adam: A Method for Stochastic Optimization. *arXiv:1412.6980 [cs]* (2017). ArXiv: 1412.6980.

9. Goodfellow, I., Bengio, Y. & Courville, A. *Deep Learning* (MIT Press, 2016). http://www.deeplearningbook.org.

10. Mitusch, S. K., Funke, S. W. & Dokken, J. S. dolfin-adjoint 2018.1: automated adjoints for FEniCS and Firedrake. *J. Open Source Softw.* **4**, 1292, DOI: 10.21105/joss.01292 (2019).

11. Kadeethum, T., Jørgensen, T. M. & Nick, H. M. Physics-informed neural networks for solving inverse problems of nonlinear biot's equations: Batch training. In *54th US Rock Mechanics/Geomechanics Symposium* (OnePetro, 2020).

12. Mathews, A. *et al.* Uncovering turbulent plasma dynamics via deep learning from partial observations. *Phys. Rev. E* **104**, 025205, DOI: 10.1103/PhysRevE.104.025205 (2021).

13. Paszke, A. *et al.* Pytorch: An imperative style, high-performance deep learning library. *Adv. neural information processing systems* **32** (2019).

14. Wu, C., Zhu, M., Tan, Q., Kartha, Y. & Lu, L. A comprehensive study of non-adaptive and residual-based adaptive sampling for physics-informed neural networks. *arXiv preprint arXiv:2207.10289* (2022).
